# Investigating colour vision and its structural correlates 15 years following a first demyelinating event

**DOI:** 10.1136/jnnp-2024-334551

**Published:** 2024-09-12

**Authors:** Charmaine Yam, Wallace J Brownlee, Ferran Prados Carrasco, Ahmed Toosy, Olga Ciccarelli

**Affiliations:** 1 Queen Square MS Centre, Department of Neuroinflammation, UCL Queen Square Institute of Neurology, University College London, London, London, UK; 2 Translational Imaging Group, Centre for Medical Image Computing (CMIC), Department of Medical Physics and Bioengineering, University College London, London, London, UK

**Keywords:** VISION, MULTIPLE SCLEROSIS, NEUROOPHTHALMOLOGY

## Abstract

**Background:**

We investigated the long-term colour and contrast vision outcomes, 15 years after a first demyelinating event, with their structural correlates using optical coherence tomography (OCT) and brain MRI.

**Methods:**

Patients recruited with their first demyelinating event, were invited~15 years later to undergo clinical assessments, OCT and brain MRI and were clinically classified according to multiple sclerosis (MS) phenotypes. Linear mixed models evaluated associations between visual outcomes, MS phenotypes and OCT measures.

**Results:**

94 patients were evaluated after a median of 14.3 years. 111 eyes affected by optic neuritis and 77 unaffected eyes were studied. Optic neuritis eyes displayed worse colour vision than unaffected eyes. Unaffected eyes showed worse colour vision in relapsing-remitting MS and secondary progressive MS (SPMS) than clinically isolated syndrome, while no similar discriminatory ability was seen for OCT measures. However, ganglion cell inner plexiform layer (GCIPL) was superior to peripapillary retinal nerve fibre layer (pRNFL) in predicting all visual outcomes. Worse colour vision was associated with lower retinal thicknesses and higher brain T2 lesion load; adding MRI volumetrics to macular GCIPL predictors did not improve model prediction of visual outcomes.

**Conclusions:**

Colour vision was impaired in unaffected eyes, especially in SPMS. GCIPL thinning underpinned this impairment more than pRNFL, suggesting neuroaxonal loss as the pathobiological substrate. The correlation between worse colour vision and increasing T2 lesion load suggests that colour dysfunction reflects overall greater disease burden. Quantitative evaluation of colour vision in addition to OCT may be useful to assess disease severity in patients after a first demyelinating event.

**WHAT IS ALREADY KNOWN ON THIS TOPIC**

**WHAT THIS STUDY ADDS**

- 15 years following a first demyelinating event, eyes unaffected by optic neuritis displayed worse colour vision in patients who developed multiple sclerosis compared with those who remained clinically isolated syndrome. Furthermore, colour vision was associated with thinner ganglion cell inner plexiform layer and peripapillary retinal nerve fibre layer and higher lesion load on MRI. Optical coherence tomography measures alone could not discriminate between different MS phenotypes, however.

**HOW THIS STUDY MIGHT AFFECT RESEARCH, PRACTICE OR POLICY**

- This study highlights the importance of quantitative evaluation of colour vision, to monitor long-term disease evolution in MS following a first demyelinating episode.

## Introduction

 Acute optic neuritis is the initial presentation in up to 25% of people with multiple sclerosis (pwMS).^1 2^ The unstratified risk of developing clinically definite MS 15 years’ after a demyelinating optic neuritis is 50% and greater if brain MRI white matter lesions are detected at baseline.^3^ The long-term outcomes in high-contrast visual acuity (HCVA) following unilateral optic neuritis were good in a 15-year follow-up study of 294 patients.^3^ An MSBase Study investigating long-term visual outcomes in patients with optic neuritis as their first demyelinating event showed that after a median follow-up period of 5.2 years patients achieved good visual outcomes, and earlier disease-modifying therapy was associated with reduced risk of visual deterioration.^4^ However, long-term outcomes for colour and low-contrast visual acuity (LCVA) in eyes affected and those remaining unaffected by optic neuritis in pwMS are lacking.

Colour vision is a marker of physical disability and neuroaxonal loss in MS, independent of optic neuritis history.^5^ 70% of pwMS without optic neuritis history exhibited colour vision impairment on the Farnsworth Munsell (FM) 100 Hue test,^6^ and this was considered to be secondary to neurodegeneration in the anterior visual pathways.^7^ An MS-VisualPath cohort of 108 relapsing-remitting MS (RRMS) or clinically isolated syndrome (CIS) patients with 1-year follow-up showed that patients with dyschromatopsia in eyes unaffected by optic neuritis, defined as ≥2 errors on Hardy-Randy-Rittler (HRR) plate testing, had a greater disability, smaller normalised brain parenchymal volume and normalised grey matter volume (NGMV) and thinner peripapillary retinal nerve fibre layer (pRNFL) thickness at baseline, compared with patients with normal colour vision.^8 9^ The HRR and Ishihara plate testing are available as screening tools for colour vision, so further evaluation of chromatic discrimination with the FM 100 Hue test is warranted.^10 11^

Low-contrast visual acuity has been shown to be correlated with Expanded Disability Status Scale (EDSS), MS functional composite score, optical coherence tomography (OCT) measures of inner retinal fibre thickness and MRI measures of disease activity, as well as having a utility as a visual outcome measure in MS clinical trials.^12^ However, like colour vision, it is a visual metric that is currently not routinely tested in clinical care and the long-term outcomes post an initial demyelinating event are not known. Exploring its role in differentiating MS subtypes, alongside colour vision, would further clarify its utility in this setting.

We, therefore, aimed to investigate long-term outcomes of colour and low-contrast vision, in addition to conventional high-contrast vision, in demyelinating disease after CIS onset and their OCT (optical coherence tomography) and MRI-related structural correlates. We hypothesised that long-term colour and low-contrast vision metrics were worse in more advanced MS (eg, secondary progressive MS (SPMS)) and were associated with OCT and MRI markers of neurodegeneration.

## Materials and methods

### Study design and participants

We analysed data from the final visit of a prospective, longitudinal, observational study of adults presenting with their first demyelinating event between 1995 and 2004 at Moorfield’s Eye Hospital or the National Hospital for Neurology and Neurosurgery.^13^ The inclusion criteria for the study were: (1) age 16–50 years, (2) a ‘typical’ clinical syndrome suggestive of MS and (3) no previous history of neurological symptoms. Other neurological disorders were excluded before inclusion in the study by the treating neurologist or neuro-ophthalmologist. 178 patients were recruited within 3 months of symptom-onset, and invited for clinical and visual assessments, OCT and MRI brain after~15 years. Each patient’s MS phenotype was classified as CIS, RRMS or SPMS (secondary progressive MS) at the most recent follow-up assessment according to Lublin *et al*.^14^

### Visual and clinical assessment

Monocular visual assessments were performed. Best corrected HCVA were assessed using logMAR Early Treatment Diabetic Retinopathy charts at 4 m. LCVA were assessed with Sloan letter charts (2.5% and 1.25% contrast) at 2 m with the score defined as the total number of letters correctly read recorded for each chart. Colour vision was assessed with the FM 100 Hue test, which tests the ability to isolate and arrange minute differences in colour targets that cover all visual Hues. The total error score (TES) was recorded, which reflects the number of tiles placed incorrectly so a higher score reflects more impaired colour vision. Patients with significant ophthalmic disease or severe refractive error (N=8) and patients with congenital colour-vision deficiency (N=2) were excluded from statistical analysis.

Medical records were reviewed with patients’ accounts to inform the history of optic neuritis history for each eye. EDSS assessments were performed in all patients by a single qualified rater (WJB).

### Optical coherence tomography acquisition and analysis

OCT images were acquired between January 2014 and December 2015 using a Spectralis SD-OCT platform (Heidelberg Engineering, Heidelberg, Germany) with software V.1.5.2.0, from both eyes per patient without pupil dilatation by a single operator (WJB). pRNFL thickness was obtained using a ring scan with a 3.4 mm diameter circle scan centred on the optic disc. A macular scan was performed with a 1, 3, 6 mm-diameter circular grid centred around the fovea. The macular ganglion cell inner plexiform layer (GCIPL) and inner nuclear layer (INL) was calculated based on average thicknesses obtained from the 3 mm diameter ring (excluding the central 1 mm ring), using inbuilt segmentation software. The quality of automated segmentation was checked by a single rater and manually corrected as necessary (CY). Significant ocular comorbidity and severe refractive error (>±6 dioptres) precluded patients from further analysis. All OCT scans acquired were quality checked and excluded if they did not meet the international OSCAR-1B consensus criteria for Retinal OCT Quality Assessment [(O) = obvious problems including violation of the protocol; (S) poor signal strength defined as < 15 dB; (C) wrong centration of scan; (A) algorithm failure; (R) retinal pathology other than MS related; (I) illumination; (B) beam placement and (R) any form of retinal pathology which may influence the OCT data] .^15^

For clinically unaffected eyes, subclinical optic neuritis eyes were defined if pRNFL was ≤75 µm, which is <2.5 SD below the weighted pRNFL mean for a healthy cohort aged between 18 and 70+ years, obtained from a Heidelberg Spectralis machine.^16^

### MRI acquisition and analysis

Participants underwent MRI brain using a 3.0 T scanner (Achieva, Phillips Healthcare Systems) at University College London Institute of Neurology, with a 32 channel receiver head coil. The following sequences were acquired: proton density/T2-weighted fast spin multi-echo scan to identify T2-hyperintense white matter lesions and a magnetisation-prepared 1 mm^3^ isotropic three-dimensional (3D) T1-weighted turbo field echo scan for tissue segmentation and volumetric derivation.

T2-hyperintense white matter lesions were manually outlined using JIM V.6.0 (JIM V.6.0, Xinapse systems, Aldwincle, UK, http://www.xinapse.com) by a single rater (WJB) to obtain T2 lesion volume (T2LV). The T2 image was co-registered to the 3D T1-weighted scan using a pseudo-T1 image generated by subtracting the proton density from the T2-weighted image.^17^ Then, the T2 hyperintense lesion masks were transformed from native space to 3D-T1 space using nearest-neighbour interpolation. The 3D T1 images were filled using a non-local patch match lesion-filling technique.^18^ Then, using Geodesic Information Flows,^19^ we performed probabilistic tissue segmentation of the lesion-filled T1-weighted scans to create cortical and deep grey matter and normal-appearing white matter masks. Brain parenchymal fraction (BPF) grey matter fraction (GMF) and white matter fraction (WMF) were calculated.

### Statistical analysis

Descriptive variables for optic neuritis and unaffected eyes are presented with medians (IQR) if non-normally distributed and with means (SD) if normally distributed. Analyses were performed using R Studio (R V.4.1.2). The type I error threshold was set at p<0.05 for statistical significance.

The following long-term visual outcomes were investigated separately—logMAR HCVA, LCVA 2.5%, LCVA 1.25%, FM TES. Square root transformation of FM error was performed to improve the normality of residuals. Linear mixed models (nlme package in R) were specified, accounting for within-subject inter-eye correlations and evaluated the associations between optic neuritis status, MS phenotypes and long-term visual outcomes. Post hoc pairwise comparisons between marginal means were performed with Benjamini-Hochberg (false discovery rate (FDR)) adjustment for multiple comparisons.^20^ Further linear mixed models investigated associations between OCT (pRNFL, macular GCIPL (mGCIPL) and INL) and MRI metrics (T2LV, BPF, WMF, GMF) as predictors, in turn and in combination, for each visual outcome. Individual participants were modelled as random intercepts.^21^ All model fixed factors included age, sex, history of optic neuritis and MS phenotype. Interactions between MS phenotype, optic neuritis and OCT metrics were included in all models. We defined an acceptable VIF (variance inflation factor) level as <5 when considering the risk of multicollinearity in our models.^22^

Non-nested models were compared using corrected Akaike information criterion (AICc) and AICc weight,^23^ accounting for smaller sample sizes according to the number of independent variables (AICcmodavg package in R) with maximum likelihood rather than REML estimation. Reported final models were estimated using restricted maximum likelihood (REML).

Finally, we determined whether the best OCT and MRI markers together were superior to OCT alone at predicting the visual outcomes by log-likelihood ratio tests comparing appropriately nested models.

## Results

Out of 178 patients studied at baseline, 166 were assessed clinically after about 15 years, as previously reported^13^; 94 patients underwent OCT, MRI, clinical and visual assessments and were included into this study. 78 of 94 patients (83%) had optic neuritis as their initial event, and an additional 3 patients (totalling 81 (86%)) experienced at least one episode of optic neuritis during follow-up (table 1). In addition, 10 subclinical optic neuritis eyes were identified. A total of 188 eyes were studied: 111 had been affected by clinical or subclinical optic neuritis and 77 remained unaffected.

**Table 1:** Demographics, clinical and MRI characteristics of patients at 15 years follow-up from the first demyelinating event

	PatientsN=94
Age at 15-year follow-up (years) (mean, SD)	47.8 (7.8)
EDSS (median, IQR)	1.50 (1.0–3.0)
Number of female patients	65 (69%)
Length of follow-up (years) (median, IQR)	14.29 (12.9–16.9)
Clinical characteristics	
Patients with optic neuritis at onset	78 (83%)
Patients developed optic neuritis during follow-up	3 (3%)
Patients with clinically unilateral optic neuritis	61 (65%)
Patients with clinically bilateral optic neuritis*	20 (21%)
Patients who did not develop optic neuritis in either eye at follow-up	13 (14%)
Subclinical optic neuritis eyes	10 (5.3%)
Total optic neuritis eyes at follow-up	111 (59%)
Total unaffected eyes at follow-up	77 (41%)
Diagnosis at follow-up	
CIS	18 (19%)
RRMS	63 (67%)
SPMS	13 (14%)
Diagnosis other than MS	0 (0%)
Time to CDMS† (months)	29.4 (7.47–54.47)
On DMT at follow-up ‡	23 (24%)
Brain MRI	
T2 lesion volume (median, IQR)	3.97 mL (0.94–14.22)
Brain parenchymal fraction (mean, SD)	0.75 (0.043)
Grey matter fraction (mean, SD)	0.46 (0.030)
White matter fraction (mean, SD)	0.28 (0.022)

* Bilateral optic neuritis denotes patients with a history of optic neuritis in both eyes inat any point in time (not simultaneous or bilateral sequential).

† Clinically definite Mmultiple Ssclerosis (CDMS).

‡ Disease modifying therapy (DMT)

CIS, clinically isolated syndrome; EDSS, Expanded Disability Status Scale; MS, multiple sclerosis ; RRMS, relapsing-remitting MS ; SPMS, secondary progressive MS.

At median 14.3 years follow-up, patients demonstrated low levels of physical disability (median EDSS 1.5) and most had RRMS (table 1). Other details of the original long-term follow-up cohort were previously provided.^13^

### Differences in visual outcomes based on optic neuritis status and between MS phenotypes

**Figure 1:**
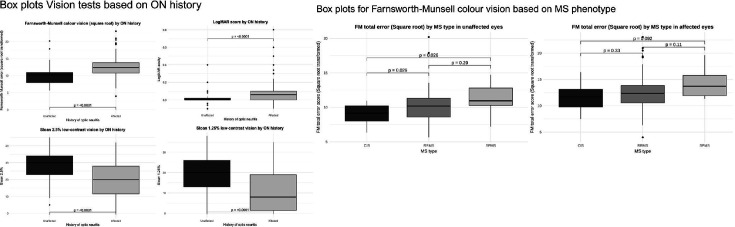
Box plots showing the relationship between vision and optic neuritis status and MS phenotype. MS, multiple sclerosis. ON, optic neuritis.

Optic neuritis eyes displayed worse visual outcomes than unaffected eyes at long-term follow-up, adjusting for age, sex and MS phenotype (figure 1). Explicitly, optic neuritis eyes demonstrated worse colour vision than unaffected eyes (√FM TES difference +2.21 units (SE 0.42) p<0.0001). Similarly, optic neuritis eyes showed worse low and high contrast visual acuity than unaffected eyes (Sloan 2.5%: difference −9.17 letters (SE 1.21) p<0.0001; Sloan 1.25%: difference −8.0 letters (SE 0.97) p<0.0001; logMAR: difference +0.07 points (SE 0.017) p<0.0001)).

Unaffected eyes exhibited worse colour vision in RRMS compared with CIS (√FM TES difference +1.96 units (SE 0.81) p=0.026) and in SPMS compared with CIS (√FM TES difference +3.02 units, (SE 1.13), p=0.026), although, for optic neuritis eyes differences between MS types were not significant (figure 1). No differences were seen between MS phenotypes in high or low-contrast acuities for optic neuritis or unaffected eyes. Summary statistics for visual outcomes in the optic neuritis (ON) and non-optic neuritis (NON) eyes, according to disease phenotype, are shown in table 2.

**Table 2:** Visual outcomes and OCT outcomes in optic neuritis eyes and in unaffected eyes according to MS phenotype

Visual outcomes	CIS (median (IQR))	RRMS (median (IQR))	SPMS (median (IQR))
Optic neuritis eyes - total FM error	136.0 (96 to 172)	152.0 (111 to 186)	188.0 (142 to 248.5)
Unaffected eyes - total FM error	84 (64 to 104)	104 (74 to 134)	120 (106 to 164)
Optic neuritis eyes - Sloan 2.5%	24.50 (21.0 to 29.0)	17.5 (11.25 to 28)	20.0 (5.0 to 24.0)
Unaffected eyes - Sloan 2.5%	29.0 (24.25 to 34.0)	30.0 (22.0 to 34.25)	27.0 (24.0 to 35.0)
Optic neuritis eyes - Sloan 1.25%	14.0 (5.50 to 20.50)	7.0 (2.0 to 18.0)	8.0 (0.0 to 19.0)
Unaffected eyes - Sloan 1.25%	15.0 (13.0 to 26.25)	20.0 (13.0 to 25.0)	21.0 (14.0 to 26.5)
Optic neuritis eyes - logMAR	0.0 (0.0 to 0.015)	0.1 (0.0 to 0.14)	0.0 (0.0 to 0.10)
Unaffected eyes - logMAR	0.0 (0.0 to 0.015)	0.0 (−0.06 to 0.02)	0.0 (0.0 to 0.02)

OCT measures (mean, SD)	CIS	RRMS	SPMS
Optic neuritis eyes - GCIPL	69.09 (16.03)	68.0 (13.98)	61.06 (10.76)
Unaffected eyes - GCIPL	89.89 (7.31)	85.36 (11.28)	79.2 (10.99)
Optic neuritis eyes - pRNFL global	76.81 (14.77)	74.26 (15.37)	71.53 (9.74)
Unaffected eyes - pRNFL global	96.32 (10.09)	90.41 (10.32)	87.09 (9.89)

CIS, clinically isolated syndrome; FM, Farnsworth Munsell; GCIPL, ganglion cell inner plexiform layer; MS, multiple sclerosis; OCT, optical coherence tomography; pRNFL, peripapillary retinal nerve fibre layer; RRMS, relapsing-remitting MS ; SPMS, secondary progressive MS.

### Differences in OCT measures (pRNFL and GCIPL) between optic neuritis and unaffected eyes and between MS phenotypes

**Figure 2:**
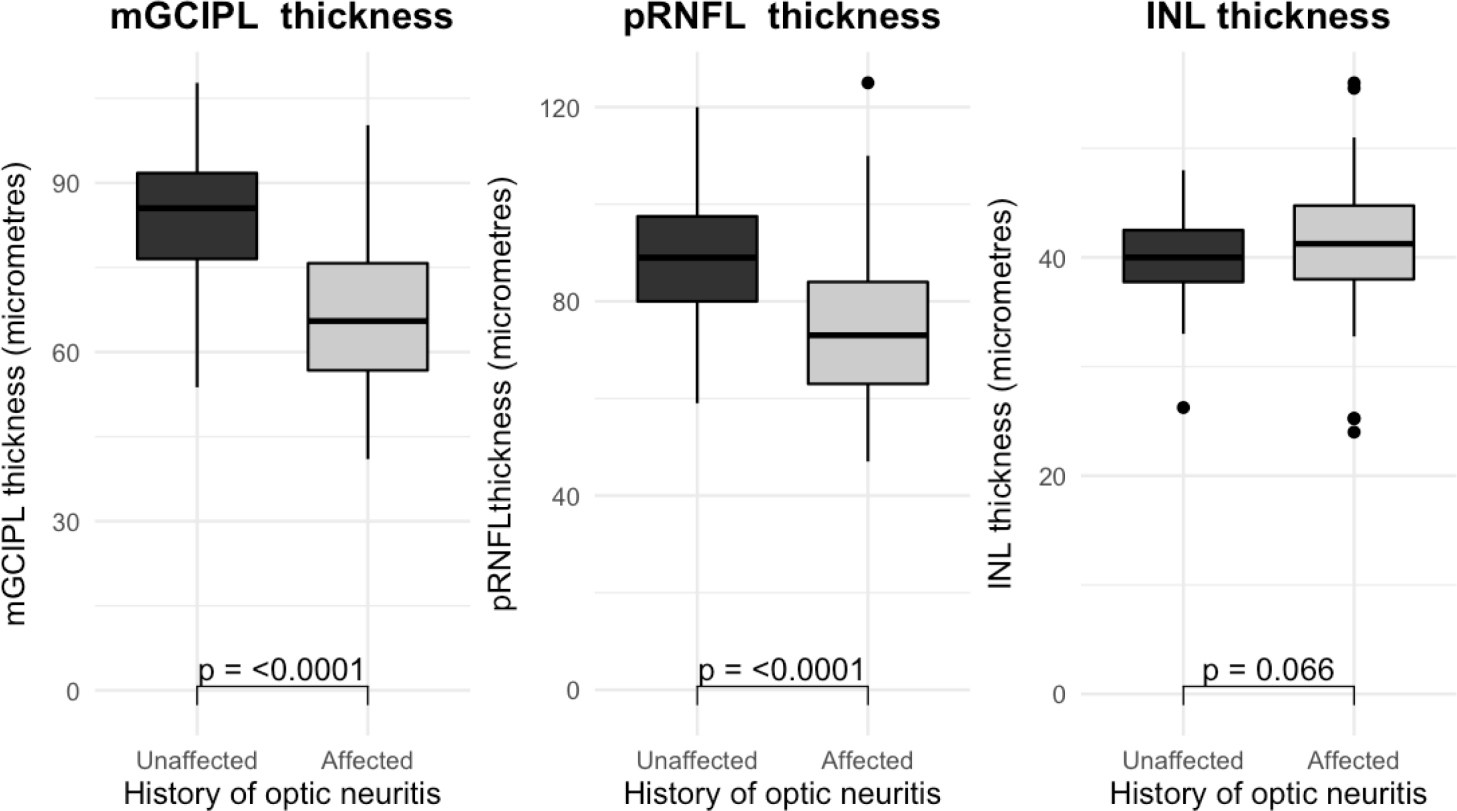
Box plots for inner retinal thickness based on optic neuritis history. INL, inner nuclear layer; mGCIPL, macular ganglion cell inner plexiform layer; pRNFL, peripapillary retinal nerve fibre layer.

At follow-up, optic neuritis eyes had thinner mGCIPL and pRNFL compared with unaffected eyes after adjusting for age, sex and MS phenotype (GCIPL: estimated means 67.0 µm (SE 1.67) vs 84.3 µm (SE 1.74) p<0.0001; pRNFL: estimated means 74.3 µm (SE 1.72) vs 89.2 µm (SE 1.78) p<0.0001) (**figure 2**). No differences in INL thickness between MS phenotypes and affected versus unaffected eyes were seen (figure 2).

Unaffected eyes showed borderline significant thinner mGCIPL in SPMS compared with CIS (difference −11.45 µm (SE 4.74), p=0.053 SPMS vs CIS) but no significant difference between RRMS and CIS or between SPMS and RRMS. Optic neuritis eyes did not show differences in OCT measures between MS phenotypes. Also, no differences were seen for pRNFL measures between MS phenotypes in either optic neuritis or unaffected eyes. The summary statistics for mGCIPL and pRNFL in the optic neuritis and unaffected eyes and according to disease subtype are shown in table 2.

### Associations between OCT measures and visual outcomes

All eyes together, worse colour vision was associated with lower mGCIPL and pRNFL thicknesses; for every 1 µm reduction in mGCIPL and pRNFL thickness there was an increase in √FM TES score by 0.11 (95% CI 0.046 to 0.17, p=0.001) and 0.094 units (0.028 to 0.16, p=0.006), respectively.

Similar associations were found between worse LCVA and thinner mGCIPL; for every 1 µm reduction in mGCIPL there was a reduction in the Sloan 2.5% score by 0.22 (95% CI 0.02 to 0.42, p=0.032) and a reduction in the Sloan 1.25% score by 0.19 (95% CI 0.017 to 0.36, p=0.031), respectively. There was no significant association between pRNFL thickness and Sloan LCVA after adjusting for interactions between MS phenotypes and pRNFL. No association was found between logMAR and mGCIPL or pRNFL thicknesses. Non-nested model comparisons indicated that mGCIPL models were superior to pRNFL in predicting all visual outcomes.

**Figure 3:**
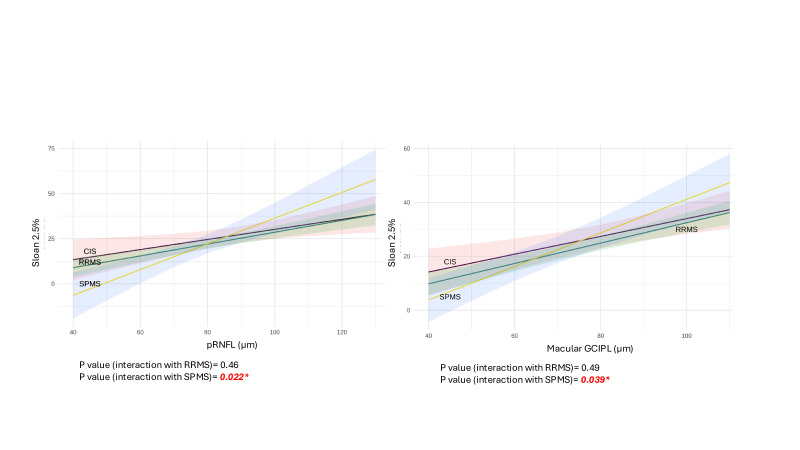
Interaction plot for optical coherence tomography in predicting Sloan 2.5%. CIS, clinically isolated syndrome; GCIPL, ganglion cell inner plexiform layer; MS, multiple sclerosis; SPMS, secondary progressive MS; RNFL, retinal nerve fibre layer; RRMS, relapsing-remitting MS.

Significant interaction effects for LCVA were seen (figure 3). Associations between Sloan 2.5%, and both pRNFL and mGCIPL were steeper in SPMS than CIS/RRMS.

### Associations between MRI and visual outcomes

Increasing T2 lesion volumes were associated with worse colour vision; for every 1 mL increase in T2 lesion volume, there was an increase (worsening) in √FM TES score by 0.067 units (95% CI 0.0075 to 0.13, p=0.028) but not with other visual outcomes. Decreasing brain volume was related to worse HCVA (increasing logMAR); for every 1% reduction in BPF there was an increase in logMAR units by 0.70 (95% CI 0.080 to 1.31, p=0.027), after adjusting for age, sex, MS phenotype and optic neuritis status. No significant associations were found between brain volumes or T2 lesion load and LCVA. Summary statistics of MRI brain metrics are shown in table 1. The associations between brain metrics (BPF, GMF, WMF and T2LV) and visual outcomes, adjusted for age, gender, MS phenotype and optic neuritis status are shown in table 3.

**Table 3:** Linear mixed models reflecting the change in visual outcomes based on the change in BPF, GMF and T2LV

Visual test	Slope (β)	SE	95% CI	P value
√FM error				
T2LV	0.066	0.03	0.0064 to 0.13	0.031*
BPF	−7.91	7.55	−22.93 to 7.11	0.29
GMF	−5.95	11.61	−28.24 to 17.63	0.61
WMF	12.6	14.86	−17.12 to 41.73	0.40
Sloan 2.5%				
T2LV	0.085	0.11	−0.13 to 0.30	0.43
BPF	40.7	26.61	−12.17 to 93.56	0.13
GMF	63.19	41.75	−19.8 to 146.18	0.13
WMF	26.55	54.63	−82.03 to 135.13	0.63
Sloan 1.25%				
T2LV	−0.047	0.11	−0.26 to 0.17	0.66
BPF	20.64	26.53	−32.07 to 73.34	0.44
GMF	8.54	41.7	−74.35 to 91.42	0.83
WMF	15.8	54.55	−92.63 to 124.22	0.77
LogMAR				
T2LV	0.00076	0.0013	−0.0017 to 0.0033	0.55
BPF	−0.70	0.31	−1.31 to −0.080	0.027*
GMF	0.88	0.49	−1.85 to 0.084	0.073
WMF	−0.010	0.64	−1.27 to 1.25	0.99

Models corrected for age, sex, history of optic neuritis and MSmultiple sclerosis phenotype.

* denotes a statistical significance p-value <0.05

BPF, brain parenchymal fraction ; GMF, grey matter fraction; T2LV, T2 lesion volume ; WMF, white matter fraction.

When we considered the most predictive MRI measures for each visual outcome combined with GCIPL as an additional predictor, model performance did not improve, using log-likelihood ratio testing.

## Discussion

15 years after a first demyelinating event, which was optic neuritis in 83% of patients, colour vision was worse in the affected eyes than unaffected eyes. Interestingly, in the unaffected eyes it was worse in patients who developed MS, especially SPMS, compared with CIS. Among the long-term visual outcomes studied at long-term follow-up, only colour vision of unaffected eyes was significantly different between MS phenotypes. The mechanisms of colour vision impairment in all eyes could be attributed to inner retinal thinning, especially GCIPL and was associated with higher brain T2 lesion load.

The long-term visual outcomes following the first demyelinating event are of interest as potential clinical markers of disease progression.^12^ Colour vision, measured with the FM 100 Hue test, has been proposed as a potential marker of neurodegeneration with predilection for involvement of the ventral occipitotemporal cortex, as a neural correlate for colour processing.^24^ Post-mortem examinations of MS brains showed preferential injury to the parvocellular visual system of the thalamus rather than the magnocellular system compared with healthy controls.^25 26^ The parvocellular system is responsible for colour and high-contrast vision, whereas magnocellular system is responsible for motion and contrast sensitivity (achromatic vision).^27^ Findings of reduced lateral geniculate nucleus volumes in patients with RRMS compared with matched healthy controls suggest a significance of this structure in MS pathology, connecting the anterior to the posterior visual pathway, hence subject to both anterograde and retrograde degeneration from the retina and optic radiation lesions, respectively.^28 29^ On the other hand, Anssari *et al* found selective loss of blue−yellow colour vision in early MS but not red−green colour vision, however both were affected in established patients with MS without optic neuritis.^30^ It is hypothesised that the cause of this difference is due to neurodegeneration, since the red–green and blue–yellow colour vision pathways are only distinctly separated at the level of the photoreceptors and the retina. This illustrates the complex pathophysiology of colour vision loss in MS with multiple areas of the visual pathway and central nervous system implicated, suggesting a diffuse process.^31^

Significantly, our study’s findings suggest that FM 100 Hue colour vision is more sensitive than HCVA or LCVA in unaffected eyes at distinguishing SPMS and RRMS from CIS many years after the first demyelinating event, supporting the proposition that colour vision is a sensitive marker of disease severity. Thinner GCIPL thicknesses were also seen in patients with SPMS with borderline significance compared with CIS in keeping with previous studies^32^,^34^ and were associated with worsening metrics for LCVA and FM 100 Hue colour vision. pRNFL was only significantly associated with colour vision and there were no significant differences between MS phenotypes, suggesting that mGCIPL is a more reliable OCT marker of disease progression following a first demyelinating episode than pRNFL. This is also consistent with previous studies correlating OCT with brain atrophy^35^ and investigating different MS subtypes.^36^ We evaluated model comparisons of mGCIPL with pRNFL which also support this conclusion, with effects seen predominantly in unaffected eyes. This study further highlights the limitations of tracking pRNFL alone as a neurodegenerative marker and inclusion of GCIPL in the routine evaluation of a patient with MS is recommended. Our study shows that FM 100 Hue colour vision can discriminate MS phenotypes and while this may, at least in part, be biologically mediated by mGCIPL, the discriminatory ability was stronger for colour vision. No effects were seen with INL on visual outcomes, which has been shown to increase during increased inflammatory activity and relapses in MS.^37^ The association between FM error with GCIPL and pRNFL but not INL further supports the role of colour vision as a neurodegenerative marker, rather than reflecting acute inflammatory activity in the CNS.

Interestingly, for MRI markers, FM 100 Hue colour vision was significantly associated with T2LV, likely to reflect more advanced disease but only weakly associated with brain volumes (table 3), which is perhaps surprising considering the association between colour vision and connections with the thalamus^27^ and the temporal differences in grey matter atrophy between MS phenotypes.^38^ HCVA logMAR however, demonstrated significant associations with BPF and borderline associations with GMF, consistent with previous findings by Wu *et al*.^39^ Martinez-Lapiscina *et al* found that HRR-related dyschromatopsia (a faster colour vision test to administer than FM 100 Hue test) correlated with a greater decrease in NGMV (normalised grey matter volume)^8^ but not with relapse frequency nor number of Gd-enhancing lesions. HRR plates have an advantage over Ishihara plates in that tritan colour vision is tested in addition to red–green colour, but a limitation is that it is more difficult for individuals with low acuity.^9^ The choice of colour vision test has implications on which structural pathway is examined and how this relates to OCT and MRI markers. This study provides evidence that FM 100 Hue colour testing, which is a cost-effective test, can discern between CIS and MS phenotypes at long-term follow-up following the first demyelinating event. It could be considered as a promising colour vision biomarker in patients with prognosticating CIS/MS patients but may need evaluation against other colour vision tests in this context.

In contrast, LCVA was not associated with any MRI metrics in our study. Previous studies have shown significant correlations between LCVA and MRI brain volumetrics.^40^ Thus, our study shows some variability in the significant associations between MRI metrics and visual outcomes, although the directions of associations are biologically consistent for brain volumes. This may, in part be explained by the relatively high proportion of optic neuritis eyes (83%) and potential floor effects of low contrast charts, which is an important limitation of this test.^41^

Our post hoc analysis showed that adding MRI metrics to GCIPL did not improve the predictive models for visual outcomes compared with entering GCIPL alone. However, OCT offers the additional practical benefits over MRI of lower costs, more convenient accessibility where available, is reproducible with fast acquisition time and can provide objective measurements in a timely manner.

Our study has several limitations. Baseline visual outcome data were not available so any longitudinal changes in vision could not be determined. The number of optic neuritis episodes was not available for each individual; a small number of patients (n=3) developed optic neuritis after their initial demyelinating event but when this event occurred during the follow-up was not corrected for in the cross-sectional analyses. Furthermore, some of the relapses including recurrent optic neuritis could have been missing as relapse data was collected prospectively at 15 years. This may account for the relatively low proportion of optic neuritis occurring during follow-up as opposed to the first demyelinating event. As the patients were diagnosed with MS at varying time points during the 15 years’ follow-up, the timing of disease modifying therapy (DMT) commencement varied due to the changing treatment landscape and guidelines, so due to this heterogeneity the effects of DMT were not evaluated in this study. The number of optic neuritis eyes was high, which may be because a significant number of participants were recruited from a specialist eye hospital where presentations were mainly optic neuritis as the first event. Expansion to a cohort containing proportionally more patients without optic neuritis could increase the power of our findings regarding unaffected eyes. Additionally, to avoid multicollinearity influences, we separated, where possible, optic neuritis status from OCT variables as predictors. However, we still account for interactions between MS phenotypes and optic neuritis status or MS phenotype and OCT metrics in our model structures. Finally, the number of patients with SPMS (although reflective of the relative proportion of progressive patients in a CIS/MS cohort) was relatively small in our cohort, limiting the generalisability of our findings to this particular subgroup.^42^

In conclusion, our study showed that colour vision exhibits differences between patients who develop MS at 15 years follow-up compared with those remaining CIS for unaffected eyes, suggesting more severe colour vision impairment could be indicative of disease conversion and progression. Furthermore, colour vision was associated with OCT-related inner retinal thinning, especially mGCIPL. MRI parameters were associated with visual outcomes: colour vision was most associated with T2 lesion load, whereas logMAR was associated most with brain volume. Our findings suggest complementary pathophysiological mechanisms leading to visual loss in MS and supports the importance of the quantitative evaluation of colour vision, in addition to low and high contrast vision, to monitor disease evolution in MS.

## Data Availability

Data are available upon reasonable request.
